# Systematic review of the use of prescription and non-prescription psychotropic drugs and their relation with mental health in university population

**DOI:** 10.3389/fpsyg.2025.1661844

**Published:** 2025-10-15

**Authors:** Catalina Espitia-Cepeda, Bárbara González-Amado, Salvador Simó-Algado, Víctor José Villanueva-Blasco

**Affiliations:** ^1^Institute for Research and Innovation in Life and Health Sciences in Central Catalonia, Universitat de Vic—Universitat Central de Catalunya (UVic-UCC), Barcelona, Spain; ^2^Faculty of Health Sciences, Valencian International University, Valencia, Spain

**Keywords:** systematic review, non-medical prescription, psychotropic drugs, stress, anxiety, depression, suicidal ideation, PRISMA

## Abstract

**Background:**

This systematic review aims to analyze the relationship between prescribed and non-prescribed use of psychotropic drugs and the presence of symptoms of depression, anxiety, stress, suicidal ideation, and suicide in the university population without a clinical disorder diagnosis, considering different types of psychotropic drugs (sedatives, tranquilizers, opioids, sleep aids).

**Methods:**

Following PRISMA 2020 guidelines, a systematic search was carried out in PubMed/MEDLINE, the Web of Science, Scopus, CINAHL and PsyInfo. Registered in PROSPERO (CRD42023446068). Thirty-four quasi-experimental studies meeting ≥60% MMAT quality were included (assessed using the MMAT) were included. Data extraction considered demographic variables, mental health outcomes, types of psychotropic drugs, and prescription status.

**Results:**

The findings reveal a significant association between both prescribed and non-prescribed psychotropic drug use and symptoms of psychological distress. Non-prescribed use was more strongly linked to anxiety, stress, and depression, whereas prescribed use was more closely related to suicidal ideation and suicide. Antidepressants, anxiolytics, and opioids were most frequently involved. Women and, in several studies, LGBTQ+ students displayed higher prevalence of psychotropic use in relation to distress.

**Conclusion:**

The results emphasize the need for a preventive, contextual, and integral approach to address psychotropic drug use in university settings. Healthy Campus initiatives should reinforce awareness campaigns, promote psychosocial well-being, ensure early detection of psychological distress, and reflect critically on academic structures that may exacerbate emotional difficulties. Further research is needed from an intersectional and multilevel perspective to inform targeted interventions and institutional policy.

**Systematic review registration:**

https://www.crd.york.ac.uk/PROSPERO/view/CRD42023446068, identifier (CRD42023446068).

## Introduction

The consumption of psychotropic drugs with and without a medical prescription is considered a growing public health problem [Bouvier et al., 2019; Hulme et al., 2018; National Institute on Drug Abuse, 2018; Rougemont-Bücking et al., 2018; Schepis et al., 2018; Substance Abuse and Mental Health Services Administration (SAMHSA), 2021].

Psychotropic drugs have inhibitory effects on the central nervous system (CNS), leading people to use them with or without a prescription to alleviate various types of physical and psychological discomfort (National Institute on Drug Abuse, 2018). The consumption of psychotropic drugs has been associated with the presence of chronic pain (Garland et al., 2020; Groenewald et al., 2019; Rogers et al., 2021), psychological distress (Ponnet et al., 2015), stress (Jensen et al., 2016), post-traumatic stress disorder (Aarstad-Martin and Boyraz, 2017), anxiety (Bouvier et al., 2019; Montiel et al., 2020; Wheeler et al., 2019), depression (Bouvier et al., 2019; Bryan et al., 2021; Kedia et al., 2020; Montiel et al., 2020; Pontes et al., 2021; Villanueva-Blasco et al., 2022b; Wheeler et al., 2019), suicidal ideation and suicide (Montiel et al., 2020; Pontes et al., 2021; Villanueva-Blasco et al., 2022b), or sleep disorders (Alasmari et al., 2022).

In recent decades, various systematic reviews and meta-analyses have been conducted on the consumption of psychotropic drugs among the general population, adolescents, young adults, and university students, with different objectives. Some have focused on specific types of psychotropic drugs, such as stimulants (i.e., Bavarian et al., 2015; Benson et al., 2015), opioids (i.e., Bonar et al., 2020; Bouvier et al., 2019; Weyandt et al., 2022), or benzodiazepines (i.e., Votaw et al., 2019). Others have analyzed aspects such as the prevalence of psychotropic drug consumption (i.e., Jia et al., 2018), changes in consumption over a lifetime (i.e., Schepis et al., 2020), the main reasons for consumption (i.e., Bennett and Holloway, 2017; Drazdowski, 2016), risk and protective factors related to consumption (i.e., Lyons et al., 2019; Nargiso et al., 2015), and the origin and diversion of psychotropic drugs for non-medical use (i.e., Hulme et al., 2018). However, most of the studies included in these systematic reviews did not differentiate between prescribed (misuse pattern) and non-prescribed psychotropic drugs.

Nowadays, non-prescribed psychotropic drugs are the second most illicitly consumed psychoactive substance after marijuana (Rougemont-Bücking et al., 2018), particularly those classified as opioids, stimulants, and sedatives (Hulme et al., 2018). The use of non-prescribed psychotropic drugs can lead to various health consequences such as overdose (World Health Organization, 2023), addiction, and increased demand for addiction treatment (Yamamoto et al., 2021), polydrug use (Aarstad-Martin and Boyraz, 2017; Bakhshaie et al., 2019; Gallucci and Martin, 2015; Molloy et al., 2019; Papazisis et al., 2018), and death [Center for Disease Control and Prevention (CDCP) and National Center for Health Statistics (NCHS), 2017].

The consumption of psychotropic drugs, both prescribed and non-prescribed, can emerge as a coping strategy in response to certain psychosocial stressors (Villanueva-Blasco et al., 2022a). This relationship between psychotropic drug use and stress factors can be explained through the Transactional Model of Stress (Lazarus and Folkman, 1986). This model proposes three coping styles: (a) task-focused, seeking logical ways to solve the problem; (b) emotion-focused, aimed at assigning a new meaning to the stressor to mitigate emotional distress; and (c) avoidance-oriented, where the individual seeks distractions to avoid facing the problem. This last coping style is significantly associated with maladaptive behaviors such as drug use and the risk of addiction (Glodosky and Cuttler, 2020; Lee-Winn et al., 2018).

Several studies agree in identifying the university population as a particularly vulnerable group to experiencing stress (Glodosky and Cuttler, 2020; Zenebe and Necho, 2019). This stress can be linked to various aspects of university life and may lead to the use of psychotropic drugs as a coping strategy. The use of non-prescribed psychotropic drugs has been associated with the motivation to improve academic performance or as a cognitive enhancement method (Cook et al., 2021; Gallucci et al., 2014; Gallucci and Martin, 2015; Molloy et al., 2019; Ponnet et al., 2015; Yomogida et al., 2018; Zahavi et al., 2023), social pressure (Ponnet et al., 2015), or job uncertainty (Colell et al., 2016). Another reason for the increased use of psychotropic drugs among university students is easy accessibility (Hulme et al., 2018). Anxiety and difficulty sleeping are some of the reasons for consuming anxiolytics (Ghandour et al., 2012). While stimulants are mainly used to increase concentration, alertness, and to study. Opioids are used to relieve physical pain, sleep, and reduce anxiety (Ghandour et al., 2012).

Beyond the university population diagnosed with a clinical disorder who receive pharmacological treatment, the consumption of psychotropic drugs in the university population appears as a coping strategy for psychosocial and academic stressors. Far from pathologizing university life, the aim of this systematic review was to answer the following research questions: What is the relationship between the use of psychotropic drugs, both prescribed and non-prescribed, and the presence of symptoms of depression, anxiety, stress, suicidal ideation, and suicidal behavior among university students? Which types of psychotropic drugs are most frequently associated with indicators of psychological distress in this population? Based on these research questions, the primary objective was to analyze the relationship between the use of psychotropic drugs (prescribed and non-prescribed) and symptoms of depression, anxiety, stress, suicidal ideation, and suicidal behavior among university students. As a secondary objective, the study aimed to identify the types of psychotropic drugs most frequently associated with various indicators of psychological distress in this population. This relationship is examined by considering different categories of psychotropic substances (sedatives, tranquilizers, opioids, and sleep-inducing medications), regardless of whether they were used with or without medical prescription.

## Method

### Data sources and search strategy

A systematic review was conducted following the Preferred Reporting Items for Systematic Review and Meta-Analysis (PRISMA) guidelines (Page et al., 2021). The search was performed in the Web of Science (WoS), APA PsycInfo, CINAHL, PubMed/MEDLINE, and Scopus databases from January of 2023 to March 2025. The research protocol was registered in PROSPERO (registration code: CRD42023446068).

Various keywords were identified based on a review of previous literature and the authors’ knowledge in the field of study. Additionally, the DeCS Thesaurus was used to identify and select specific terms for the initially selected keywords. Subsequently, through an iterative process, new keywords were selected based on the results reported in the initial searches. Finally, the keywords were combined using the following search strategy: (((((((((((“College student*”) OR (“University student*”)) OR (“young adult*”)) OR (College*)) OR (“Student*”)) OR (“Emerging adulthood*”)) OR (Undergraduate*)) AND ((((“risk factor*”) OR (“relation*”)) OR (“associa*”))) AND ((((((“Anxiety*”) OR (“Stress*”)) OR (“Suicide*”)) OR (“Suicidal ideation*”)) OR (“depression*”)) OR (“Psychological distress*”))) AND (((((((((((((((((“Psychotropic drug*”) OR (“Psychotropic medication misuse”)) OR (“non-medical use of prescription drugs”)) OR (“non-medical prescription drug use”)) OR (“Prescription drug misuse”)) OR (“Hypnotic*”)) OR (“Sedativ*”)) OR (“Analgesic*”)) OR (“Opioid*”)) OR (“Substance misuse”)) OR (“Stimulant*”)) OR (“non-medical prescription opioid*”)) OR (“non-medical prescription drug*”)) OR (“opioid analgesics*”)) OR (“nonmedical use of prescription drugs”)) OR (“nonmedical prescription drug”)))).

### Eligibility criteria

The studies included in this review met the following inclusion criteria: (a) Studies with an university population sample (undergraduate and postgraduate) or that, even incorporating other types of populations, included differentiated results for the university population; (b) Studies that included quantitative data on variables of consumption with and without medical prescription of: sedatives, tranquillizers, opioids, sleeping pills; (c) Studies that analyzed the relationship between consumption and mental health variables, including depression, anxiety, stress, suicidal ideation and suicide; (d) Studies published in English and Spanish; (e) No date limit; and, (f) Minimum methodological quality of 60% (MMAT—Mixed Methods Appraisal Tool).

In addition, the following exclusion criteria were considered: (a) Studies conducted in animals; (b) University population diagnosed with mental disorders or previous addiction; (c) Studies addressing behavioral addictions; and (e) Literature reviews, systematic reviews, meta-analyses, books, book chapters, conference communications and doctoral theses.

### Selection process

Three authors (first author, second author, and fourth author) independently identified the sought studies in three steps following the literature (Gunnell et al., 2020). First, the titles of articles obtained from the initial searches were examined and selected based on the eligibility criteria mentioned earlier. Next, a review of titles and abstracts was conducted to select articles that aligned with the review objectives. Third, full-text articles were thoroughly analyzed and selected for eligibility. Lastly, the bibliographic references of all selected articles were manually reviewed to identify relevant articles missed in the initial search strategy (ancestry approach).

**Figure 1:**
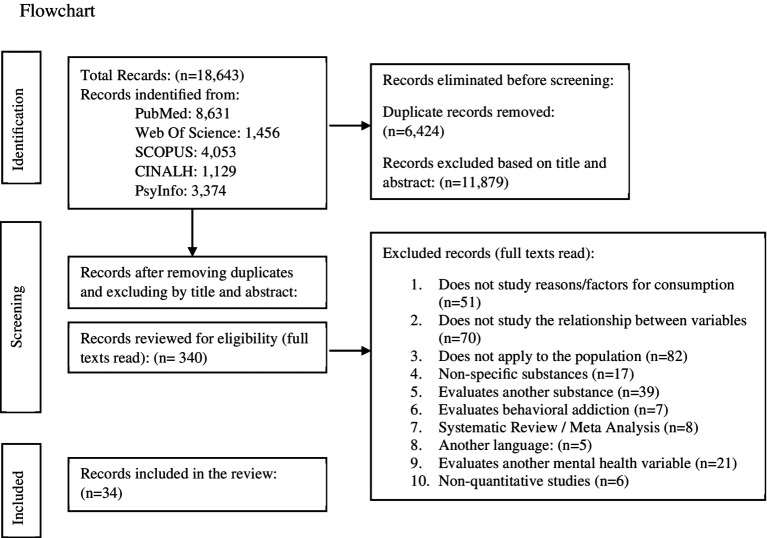
Flowchart. The flowchart was created using the PRISMA tool (Page et al., 2021).

The search strategy yielded a total of 18,643 records and 8 articles listed in the references of others articles, with 34 articles remaining after the entire selection process that were included in the systematic review. The selection process is summarized in Figure 1.

### Data extraction

Two authors (first author, and fourth author) independently and systematically extracted data from the final list of included studies. The following categories of manuscripts were identified and considered: (a) Authorship, year and country, (b) Study type, (c) Characteristics of the target population: sex and age, (d) Sample size, (e) Study objectives, (f) Mental health variables, (g) Mental health assessment (h) Psychotropic drug variables, (i) Psychotropic drug assessment, and (j) Main study outcomes. Discrepancies between the authors were resolved through consensus decision-making.

### Assessment of methodological quality

The methodological quality of the studies was assessed using the Mixed Methods Appraisal Tool (MMAT) (Hong et al., 2018). The MMAT is a critical appraisal tool designed for systematic reviews that include quasi-experimental empirical studies (Table 1). For this study, only studies providing quantitative data were included. The assessment of methodological quality for each study is presented in Table 1. A decision was made to select studies with a minimum of 60% methodological quality.

**Table 1:** Assessment of methodological quality for quasi-experimental studies.

Reference	P1	P2	P3	P4	P5	% of compliance
Freibott et al. (2024)	Yes	Yes	No	Yes	Yes	80%
Gaume et al. (2024)	Yes	Yes	Yes	Yes	Yes	100%
Kouros and Papp (2024)	Yes	Yes	No	Yes	Yes	80%
Bahlaq et al. (2023)	Yes	Yes	No	Yes	Yes	80%
Hua et al. (2023)	Yes	Yes	Yes	Yes	Yes	100%
Papp et al. (2023)	Yes	Yes	Yes	Yes	No	80%
Čanković et al. (2023)	Yes	Yes	Yes	Yes	Yes	100%
Antshel et al. (2021)	Yes	Yes	No	Yes	No	60%
Schepis et al. (2021)	Yes	No	Yes	Yes	Yes	80%
Weyandt et al. (2021)	Yes	Yes	No	Yes	Yes	80%
Davis et al. (2020)	No	No	Yes	Yes	Yes	60%
King et al. (2020)	Yes	Yes	Yes	Yes	No	80%
Sousa et al. (2020)	Yes	Yes	Yes	Yes	Yes	100%
Tam et al. (2020)	Yes	Yes	Yes	Yes	No	80%
Sattler (2019)	Yes	Yes	No	Yes	Yes	80%
Pate and Bolin (2019)	No	Yes	No	Yes	No	40%
Balayssac et al. (2018)	Yes	Yes	No	Yes	Yes	80%
Grant et al. (2018)	Yes	Yes	No	Yes	No	60%
Walters et al. (2018)	Yes	Yes	No	Yes	Yes	80%
Benson and Flory (2017)	No	Yes	Yes	Yes	Yes	80%
Boulton and O'Connell (2017)	Yes	Yes	No	Yes	Yes	80%
Meshesha et al. (2017)	Yes	Yes	No	Yes	Yes	80%
Peralta et al. (2016)	Yes	Yes	No	Yes	Yes	80%
Jeffers et al. (2015)	Yes	Yes	Yes	Yes	Yes	100%
Benotsch et al. (2014)	Yes	Yes	Yes	Yes	Yes	100%
Verdi et al. (2014)	Yes	Yes	No	Yes	Yes	80%
Betancourt et al. (2013)	Yes	No	No	Yes	Yes	60%
Cabriales et al. (2013)	No	No	Yes	Yes	Yes	60%
Dussault and Weyandt (2013)	No	Yes	Yes	Yes	Yes	80%
Zullig and Divin (2012)	Yes	Yes	No	Yes	Yes	80%
McCauley et al. (2011)	Yes	Yes	No	Yes	Yes	80%
Teter et al. (2010)	Yes	No	No	Yes	Yes	60%
Vidourek et al. (2010)	Yes	Yes	Yes	Yes	Yes	100%
Weyandt et al. (2009)	Yes	Yes	Yes	Yes	Yes	100%
Ford and Schroeder (2008)	Yes	No	Yes	Yes	Yes	80%

P1: Are participants representative of the target population?; P2: Are the measurements appropriate regarding both outcome and intervention (or exposure)?; P3: Are there complete outcome data?; P4: Are confounding factors considered in the design and analysis?; P5: During the study period, was the intervention administered (or exposure occurred) as planned?

## Results

### Characteristics and results of selected studies

Table 2 presents information from the 34 articles identified with a methodological quality of 60% or higher. In terms of study design, all were quasi-experimental. Most of the studies were conducted in the USA (*n* = 25), with others in France (*n* = 1), Brazil (*n* = 1), Canada (*n* = 1), Germany (*n* = 1), Puerto Rico (*n* = 1), Saudi Arabia (*n* = 1), Switzerland (*n* = 1), China (*n* = 1) and Serbia (*n* = 1). The age of participants ranged from 17 to 58 years, considering samples not only from undergraduate students but also from master’s and doctoral programs. The sample sizes across all studies were generally large, ranging from 41 to 22,783 participants.

**Table 2:** Coding table of primary studies: articles included in the systematic review.

Autorship, year and country	Study type	Characteristics of the target population: sex and age	Sample size	Study objectives	Mental Health Variables	Mental Health Assessment	Psychotropic drug variables	Psychotropic drug assessment	Main study outcomes
Freibott et al. (2024). USA	Quantitative non-randomized	Age: 18–25 *M*_age_ = 20.52 (*SD = 1.94*) Females: 67%	*N* = 782 (opioid misuse)	(1) Quantify the prevalence of opioid misuse in a large, national sample of college students, (2) estimate the prevalence of depression and anxiety among students reporting opioid misuse, (3) document the mental health helpseeking behaviors of students reporting opioid misuse and (4) describe the academic performance of students reporting opioid misuse.	Depression Anxiety Mental health diagnosis	PHQ-9 (*α* = 0.89) GAD-7 (*α* = 0.91)	Opioid misuse	*Ad hoc* question: “Over the past 30 days, have you used any of the following drugs (select all that apply).”	Of the 782 students reporting opioid misuse, 503 (64.3%) screened positive for anxiety or depression (*p* < 0.001) and 453 (57.9%) reported a mental health diagnosis (*p* < 0.001). While 82.8% of students reporting opioid misuse indicated a need for mental or emotional help in the last 12 months, only 48.0% reported counseling or therapy in the same time frame.
Gaume et al. (2024) Switzerland	Quantitative non-randomized	Age: — *M*_age_ = −(*SD = −*) Females: -%	*N* = 141 (Non-medial use of prescription drugs)	Evaluated the prevalence of substance use among medical students, and then investigated whether mental health and burnout variables had an influence on substance use.	Depressive symptoms Suicidal ideation Anxiety Stress	CES-D (French version) Beck Depression Inventory State–Trait Anxiety Inventory	Substance use Neuroenhancement drugs, as well as non-medical use of prescription drugs	French version of the WHO’s Alcohol, Smoking and Substance Involvement Screening Test Cohort Study on Substance Use Risk Factors questionnaire	Higher scores on the factor related to sedatives, nonmedical prescription drugs, and neuroenhancement drugs use (S3) was significantly related with higher scores on the factor related to depression and anxiety (M1). The factor comprising stress related to studies and work/life balance (M3) was not associated with any substance factor
Kouros and Papp (2024) USA	Quantitative non-randomized	Age: 18–21 *M*_age_ = 19.5 (*SD = 0.71*) Females: 69%	*N* = 300	Tested associations between trajectories of PDM problems and college students’ mental health and subjective happiness over time.	Internalizing symptoms: depressive symptoms Externalizing symptoms	Brief Patient Health Questionnaire: depressive symptoms (α = 0.86–0.92) Inventory of Depression and Anxiety Symptoms, social anxiety subscale: social anxiety (α = 0.85–0.89) Externalizing Spectrum Inventory: General Disinhibition subscale assessed impulsive behavior or lack of constraint (α = 0.85–0.88)	Problems with prescription misuse Other Substance Use Problems	DAST-10 (Modified version) (α = 0.71–0.79) Rutgers Alcohol Problem Index (α = 0.80–0.87)	Problems with PDM were concurrently associated with higher levels of depressive symptoms, disinhibition, callousness/aggression, and lower levels of subjective happiness at T1. Further, we found support for parallel trajectories between PDM problems and both depressive symptoms and general disinhibition. Participants whose PDM problems were on an increasing (worsening) trajectory at baseline showed an increase in depressive symptoms and general disinhibition over the next two years.
Bahlaq et al. (2023) Saudi Arabia	Quantitative non-randomized	Age: - *M*_age_ = −(*SD = -*) Females: 51.4%	*N* = 732	Determine the prevalence, association, and predictors of burnout, stress, and stimulant abuse among medical and dental students in the Western region of Saudi Arabia	Stress	Cohen’s 10-item Self-Perceived Stress Scale (α = 0.82)	Stimulant abuse	Ad hoc question	There was a significant association between burnout and stimulant abuse, with all the eight students who used stimulants experiencing burnout. Most students with highly positive perception of stimulant abuse reported moderate stress (95.5%). There was a statistically significant trend in stress levels and burnout.
Hua et al. (2023) China	Quantitative non-randomized	*M*_age_ = 19.83 (*SD = 1.3*) Females: 69%	*N* = 1,703	Investigate (1) the possible associations of NMUPD with depressive and anxiety symptoms; (2) whether these associations vary by sex.	Depressive symptoms Anxiety symptoms	CES-D (α = 0.79) GAD-7 (α = 0.90)	Non-medical use of prescription drugs	Ad hoc question: how many times have you ever used the following medications, when you were not sick or without a doctor’s prescription?	Except for the frequent users of opioids, non- medical use of opioids (experimenters: *β* = 1.10 [95% CI, 0.62 to 1.57]) and sedatives (experimenters: β = 1.57 [95% CI, 0.84 to 2.31]; frequent users: β = 2.98 [95% CI, 0.70 to 5.26]) were significantly associated with depressive symptoms, even after controlling for multiple covariates and the comorbid symptom. The adjusted associations with anxiety symptoms were also significant for non-medical use of opioids
Papp et al. (2023) USA	Quantitative non-randomized	Age: 18–21 *M*_age_ = 19.5 (*SD =* 0.71) Females: 69%	*N* = 297 (prescription drug misuse)	Examined associations between stress intensity and prescription drug misuse in daily life among college students with elevated risk for engaging in the behavior.	Stress	Stress intensity was evaluated by the number of stressors experienced in the moment	Prescription drug misuse: Sedatives or sleeping pills, tranquilizers or anxiety medications, stimulants, and pain relievers.	Ad hoc question: ‘Are you about to take a medication listed here in any way a doctor did not direct you to use it?’ for 4 classes of medication (sedatives or sleeping pills, tranquilisers or anxiety medications, stimulants, and pain relievers) Have you recently taken a medication listed here not as prescribed?’ (past 3 months)	Participants were more likely to engage in prescription misuse in daily life in moments of their higher-than-usual stress, accounting for number of stressors they experienced in the moment (AOR = 1.084, *p* < 0.001, *d* = 0.04)
Čanković et al. (2023) Serbia	Quantitative non-randomized	Age: *M*_age_ = 20.95 (*SD=*) Females: 67.5%	*N* = 308	Evaluate the prevalence of depressive symptoms and examine the association between various risk factors and depressive symptoms	Depression Suicidal ideation	PHQ-9	Alcohol use and frequency of binge drinking Lifestyle factors: smoking, alcohol use, marijuana use, ecstasy use, sedative or sleeping pills use without a prescription	*Ad hoc*	Univariate analysis showed a statistically significant association between depressive episodes and self-assessed material status, social health, self-esteem, and use of sleeping pills or sedatives without prescription among first-year students. Among sixth-year students, the association of depressive episodes was significant with social health, self-esteem, and the use of sleeping pills or sedatives without prescription. Those who had used sleeping pills or sedatives without a prescription were more than four times more likely to have a PHQ-9 score ≥ 10 than those who had not (OR = 4.97, 95% CI: 1.83–13.52).
Antshel et al. (2021) USA	Quantitative non-randomized	Age: 18–25 *M_age_* = 18.9 (*SD* = 1.5) Females: 51.6%	*N* = 309 *n* = 38 history of stimulant misuse *n* = 271	Examined the impact of achievement goal orientation on stimulant misuse in college students, with stress as covariate	Stress	PSS (α = 0.83)	Stimulant medication misuse	History of stimulant misuse within the past 12 months (yes vs. no)	Inconsistent with previous research, stress, *F* (1, 302) = 1.10, *p* = 0.297, eta^2^ = 0.01, were comparable between stimulant misusers and non-stimulant misusers. *N*(misusers, PSS) = 20.27, SD = 5.811, *n* = 38 *N*(non-misusers, PSS) = 19.42 SD = 6.206, *n* = 271 *d* = 0.14
Schepis et al. (2021) USA	Quantitative non-randomized	Age: 18–25 *M_age_* = 20.5 (*SD* = 1.57) Females: 66%	*N* = 41 (≥ 6 past year prescription stimulant misuse episodes)	Evaluate the relationship between stress and PSM over a 21-day EMA period.	Academic stress	PSS-4	Prescription stimulant misuse	Current Prescription Stimulant Misuse (6 past-year PSM episodes), measured by ecological momentary Assessment (EMA): “Have you misused a stimulant since the last survey? That is, have you used your own stimulant medication in a way your doctor did not intend or have you used another person’s stimulant medication?”	At the daily level, PSS-4 total score and items were unrelated to PSM, Beta = 0.03, *p* = 0.539. At the current momentary level, PSS-4 total was significantly related to PSM, with decreases in global stress around the PSM episode (Beta = 0.09, *p* = 0.042).
Weyandt et al. (2021) USA	Quantitative non-randomized	Age: 18–24 *M_age_* = 20 (*SD* = 1.28) Females: 71.2%	*N* = 847 *NMPO_lifetime_ =* 92 (10.9%) *NMPO_past-month_:* = 7 (0.8%)	Prevalence lifetime and past 30-days of NMPO. To study the relationship between depression and anxiety symptoms and NMPO	Depression and anxiety symptoms.	DSM-5 Self-Rated Level 1 Cross-Cutting Symptom Measure—Adult. (α = 0.817 and α = 0.843, for depressive and anxiety symptoms, respectively).	NMPOQ Frequency of Use (lifetime and 30 past days) NMBM NMPS	Ad hoc questionnaire: - Lifetime: “have you ever used prescription opioids non-medically in your lifetime?” When answering “yes,” past-month opioid use was measured by the number of days in the past 30 days the opioids were used.	Lifetime nonmedical use of Benzodiazepine: 79 (9.3%) Lifetime Nonmedical Use of Prescription Stimulants: 160 (18.9%) Relationship between NMPO and depression symptoms: *r* = 0.167 Relationship between NMPO and anxiety symptoms: *r* = 0.081 (ns) Relationship between NMPS and depression symptoms: *r* = 0.145 Relationship between NMPS and anxiety symptoms: *r* = 0.117 Relationship between NMBM and depression symptoms: *r* = 0.126 Relationship between NMBM and anxiety symptoms: *r* = 0.126
Davis et al. (2020) USA	Quantitative non-randomized	*M_age_* = 21.7 (*SD* = 4.99) Females: 69.5%	*N* = 889 *N_Opioide Misuse_ =* 192 (21.6%)	Examining the relationship between prescription opioid misuse and suicidality (suicidal ideation, suicide planning and suicide attempts)	Three facets of Suicidality: suicidal ideation, suicide planning and suicide attempts. Psychological disorder: depression or other mental health disorder	Suicidality: using 3 items ad hoc. Psychological disorder: prior physician diagnosis.	Prescription opioid misuse (POM), other illicit and prescription drug use	Ad hoc question: “How frequently over the past 12-months have you used prescription opioid medications in a way not specifically directed by a doctor?” Response options were 1 = never, 2 = 1–2 occasions, 3 = 3–5 occasions, 4 = 6–9 occasions, 5 = 10–19 occasions, 6 = 20–39 occasions, and 7 = 40 or more occasions. The same format question for other illicit and prescription drug use	Unadjusted logistic regression models estimated the bivariate relationship between each suicidality variable and POM: suicidal ideation (*OR* = 4.85, CI: 3.44–6.84, *p* < 0.001), planning (OR = 6.57, CI: 4.27–10.13, *p* < 0.01), and attempts (*OR* = 26.95, CI: 13.78–52.71, *p* < 0.001).
King et al. (2020) Canada	Quantitative non-randomized	Age: 18–35 Females: 70.5% (*n* = 2,229)	*N* = 3,160 *N*_NMPS_ = 99 (3.1%) for staying awake	Prevalence and factors associated with non-medical use of NMPS to promote wakefulness	Anxiety and depressive symptoms.	HADS (α = 0.82 for depression and α = 0.83 for anxiety)	Non-medical prescription stimulant.	Question ad hoc: whether they used any kind of stimulant to help them stay awake (yes vs. no). Participants who answered “yes” were then asked to select all of the prescription medication they currently use or have used in the past to help them stay awake (even if they were not prescribed)	Females were significantly less likely to misuse prescription stimulants compared to males (*OR* = 0.64; 95% CI = 0.42–0.97; *p* = 0.037). Univariate analysis: Individuals classified as having depressive symptoms in the clinical range (OR = 2.89; 95% CI = 1.66–5.04; *p* < 0.001) were significantly more likely to misuse prescription stimulants to stay awake compared to individuals without symptoms of depression. Participants with clinical levels of anxiety were more than twice as likely to misuse prescription stimulants compared to participants with typical levels of anxiety (*OR* = 2.38; 95% CI = 1.43–3.95; *p* = 0.001).
Sousa et al. (2020) Brazil	Quantitative non-randomized	Age: 17–57 *M_age_* = 26.7 (*SD* = 8.0) Females: 75.2% (*n* = 137)	*N* = 182 *N*_NMPM_ = 144 (79.2%) lifetime *N*_NMPM_ = 38 (29.9%) last year	To assess the use of nonprescription psychoactive medications and their associations with health aspects among nursing students.	Depression	PHQ2	Use and frequency of psychoactive medication use without prescription last year.	Ad hoc questionnaire: question about use and frequency of psychoactive medication use without prescription last year	Non-prescription consumption group: (M = 1.95; SD = 2.03), non-consumption (M = 1.35, SD = 1.95).
Tam et al. (2020) USA	Quantitative non-randomized	Age: > 18 *M_age_* = 19.78 (*SD* = 2.83) Females: 68.7%	*N* = 1,052 N_NMUPD_ = 252 (24%) in the past three months.	Examine the relationship of perceived stress, psychiatric symptoms (depression and social anxiety), and NMUPD (opioids, sedatives, anxiolytics, and stimulants) among college students	Perceived stress, Psychiatric symptoms (depression and social anxiety).	Perceived stress: 14-item scale to measure the degree to which situations in the participant’s life were appraised as stressful. (α = 0.76) SF-CESD (α = 0.86) SSA (α = 0.94)	Non-medical use of prescription drugs (opioids, sedatives, anxiolytics and stimulants).	*Ad Hoc* questionnaire: a first question bout whether they had ever used a prescription drug without a doctor’s prescription, which ones and the number of times (their lifetime and post three months)	All four classes of NMUPD opioids correlated positively with perceived stress (*r* = 0.105) and depression (*r* = 0.110); sedatives with perceives stress (*r* = 0.090) and depression (*r* = 0.098); anxiolytics with perceives stress (*r* = 0.124), depression (*r* = 0.180) and social anxiety (*r* = 0.087); and stimulants with perceived stress (*r* = 0.076) and depression (*r* = 0.093). According to the CFA model, perceived stress was significantly correlated with NMUPD (r = 0.23; *p* < 0.001). Likewise, psychiatric symptoms were significantly and positively correlated with NMUPD (r = 0.31, *p* < 0.001).
Sattler (2019) Germany	Quantitative non-randomized	Age: > 18 Females: 61.9%	*N =* 2,203 *N_non-NMPD_* = 2,164 *N*_*NMPDs* =_ 39 (2%)	Study the association between perceived stress on selfreported Cognitive Enhacement drug use.	Perceived Stress	Perceived Stress Scale (PSS4) α = 0.85	Stimulants: Methylphenidate, modafinil, amphetamine-dextroamphetamine	*Ad hoc* question about non-medical use of prescription drugs to enhance cognitive efficiency (past 6 months)	Increasing reported chronic stress is positively correlated with the likelihood of self-reported CE-drug use *OR* = 1.747, 95% CI = 1.288, 2.370, *p* < 0.001
Balayssac et al. (2018) France	Quantitative non-randomized	Age: > 18 *M_age_* = 22 (*SD* = 2.3) Females: 65.9%	*N* = 2,575 *N_NMPD_ =* 252	Assess the prevalence of psychotropic drug use (medications and illegal drugs) by French pharmacy students, by carrying out a nationwide cross-sectional study. The relation of these medications and illegal drug use with several comorbidities and academic achievement was also assessed	Anxiety, depression and stress symptoms.	HADS: depression, anxiety and stress.	Psychotropic drug use (medications): Alprazolam, Bromazepam, Zolpidem Codeine, Tramadol, Escitalopram, Zopiclone, Paroxetine, Oxazepam, Diazepam, Fluoxetine. Indication of use, type of prescription for medication (medical or self-medication) and frequency of use.	Ad hoc questionnaire	Stress was significantly higher for self-medication psychotropic users (*M* = 62, *SD* = 19,2) contrasting to non-users (*M* = 54.3, *SD* = 23.3) (*p* < 0.05) Self-medications- anxiety: *OR* = 4.08, CI = 2.5, 6.70; *p* < 0.001 Self-medications- depression: *OR* = 5.79, CI = 2.86, 11.76; *p* < 0.001 Self-medications- stress: *OR* = 5.79, CI = 2.86, 11.76; *p* < 0.001
Grant et al. (2018) USA	Quantitative non-randomized	Age: (University sample) Females: 59.7%	*N* = 3,421 *N_NMPS_* = 230 (currently misuse stimulants) *n*_NMPD_ = 199 (misused stimulants lifetime). *n* = 984 never misuse stimulants	Examine the occurrence of the nonmedical use of prescription stimulants (amphetamines and methylphenidate) in a university sample and their associated physical and mental health correlates.	Depression, anxiety.	Patient Health Questionnaire (PHQ-9): depressive symptoms. Generalized Anxiety Disorder 7 (GAD-7): generalized anxiety disorder.	Prescription stimulants or amphetamines	Ad hoc question (“Please mark the frequency with which you have used prescription stimulants or amphetamines within the past 12 months. DO NOT include drugs prescribed for you.”)	Nonmedical use of stimulants was significantly associated with anxiety symptoms (χ^2^_(6)_ = 21.62, *p* = 0.001); but not with depression symptoms (*F*(2, 1,350) = 4.252; *p* = 0.014)
Walters et al. (2018) USA	Quantitative non-randomized	Age: 18–25 *M*_age_ = 20.73 (*SD = 1.61*) Females: 70%	*N* = 891	Test associations between anxious and depressive symptoms and substance use (i.e., alcohol, cannabis, tobacco, cocaine, other amphetamines, sedatives, hallucinogens, and designer drugs).	Anxiety and depressive symptoms	Personality Assessment Inventory-6	Substance use: alcohol, cannabis, tobacco, cocaine, other amphetamines, sedatives, hallucinogens, opiates, inhalants, designer drugs and steroids.	CORE Alcohol and Drug Survey – short	Depressive symptoms were associated with use of cannabis, tobacco, amphetamines, cocaine, sedatives, and hallucinogens. Anxiety symptoms were unrelated to substance use.
Benson and Flory (2017) USA	Quantitative non-randomized	Age: 18–26 *M_age_* = 20 (*SD* = 1.4) Females: 76%	*N* = 890 *n*_MISUSERS_ *=* 205 (23%) with prescribed medication. *n*_MISUSERS_ *=* 164 (18%) without any prescription for stimulant medication	Analyze the relationship between symptoms of depression and misuse of stimulant medication	Depression	CESD-R. α = 0.91	Stimulant medication use	Ad hoc questionnaire about past 12-months stimulant drug misuse	Symptoms of depression were significantly related to stimulant medication misuse. The odds of misusing increased by 1.02 (95% CI = 1.01–1.04; *p* = 0.001) for every one-point increase in depression.
Boulton and O'Connell (2017) USA	Quantitative non-randomized	Age: 17–58 Women: 93%	*N* = 4,033 *n*_NMPD_ = 408 (past year) Nursing students	Examined whether stress and perceived faculty support were related to substance misuse.	Stress	Stress: SNSI. α = 0.89	Substance use: nonprescribed prescription drugs	Personal use survey: analyze nonprescribed prescription drugs in the past year	For every 10-point increase in stress scores, students were 1.17 times more likely to report nonprescribed drug use than those students with lower scores on the SNSI (OR = 1.17, *p* < 0.001).
Meshesha et al. (2017) USA	Quantitative non-randomized	*M_age_* = 20.01 (*SD* = 1.6) Females:62%	*N* = 71 *n_NMPO_* = 35 (past year); *n* = 36 (control participants)	Evaluate the behavioral economic hypotheses that NMPO use would be associated with lower levels of reinforcement from substance-free activities and future time orientation.	Anhedonia, Depression	DASS-21: depression and anxiety	Non-medical use of prescription drugs.	Clinical interview to assess nonmedical use of prescription drugs past-year.	The NMPO group reported higher depression, [*t* (69) = 3.99, *p* < 0.001], compared to the control group.
Peralta et al. (2016) USA	Quantitative non-randomized	Age: 18–25 *M_age_* = 19.6 Females: 60.4%	*N =* 796 *n*_NMUPD_ = 236	H1: Males will have higher-odds of Non-Medical Use of Prescription Drugs (NMUPD) compared to females	Depression	CES-D: depression	Non-medical use of prescription drugs: sedatives, tranquilizers, narcotics, steroids	Ad hoc question (example: “On how many occasions (if any) have you taken *tranquilizers* on your own—that is, without a doctor telling you to take them …”)	In Model 1, age, sex, race and depression all have a positive, significant influence on NMUPD (OR_CES-D_ = 1,08; *p* < 0.001).
Jeffers et al. (2015) USA	Quantitative non-randomized	Age: 18–25 *M_age_* = 18.8 (*SD* = 1.2) Females: 64.6%	*N* = 758 *n*_NMUPD =_ 225 (29.7%) Lifetime *n*_NMUPD =_ 145 (19.1%) past 3 months	Examine the relations among health anxiety, NMUPD, and other psychological variables related to substance use	Anxiety, depression, and somatic distress.	BSI-18: anxiety, depression and somatic distress. α = 0.94	Non-medical Prescription Drugs: Analgesics, anxiolytics, stimulants, and sedatives.	Ad hoc question	Participants who reported NMUPD lifetime, had higher scores in anxiety (*t* = −2.59, *p* < 0.05), depression (*t* = −3.42, *p* < 0.01) and somatic distress (*t* = −2.04, *p* < 0.01). Likewise, Participants who reported NMUPD in the past 3 months, had higher scores in anxiety (*t* = −2.40, *p* < 0.01) and depression (*t* = −3.11, *p* < 0.01). At a multivariable logistic regression health anxiety was a risk factor for NMUPD (OR = 1.03, CI = 1.002, 1.06, *p* < 0.05), and predicted NMUPD over and above other variables.
Benotsch et al. (2014) USA	Quantitative non-randomized	Age: 18–25 *M_age_* = 18.9 (*SD* = 1.4) Females: 63%	*N* = 767 *N*_OTC_ = 100 (13%)	Examine associations between the misuse of OTC medications and psychological variables (anxiety, depression and somatic distress)	Anxiety, depression	Brief Symptom Inventory (BSI-18): depression, anxiety and somatic distress α = 0.94	Non-medical use of over-the-counter (OTC). Non-medical use of Prescription stimulants, analgesics, anxiolytics and sedatives, lifetime and in the previous 3 months	Ad hoc questionnaire	Participants who indicated they misused OTC medications scored higher in depression (*t* = 4.87, *p* < 0.001) and anxiety (*t* = 5.50, *p* < 0.001) than those who did not.
Verdi et al. (2014) USA	Quantitative non-randomized	Age: 22–29 (65.8%) Females: 72.1%	*N =* 807 *n*_nonmedical_ = 141 (17.5%) – lifetime *n*_nonmedical =_ 48 (5,9%) – past year	Examine graduate students’ non-medical use of prescription stimulant medication, and the relationship between non-medical use of prescription stimulants with psychological factors (i.e., anxiety, depression, and stress), and internal restlessness	Anxiety, depression, and stress	DASS-21. Depression (α = 0.89), Anxiety (α = 0.76), and Stress (α = 0.88; α = 0.87).	Non-medical use of prescription stimulant	SSQ (α = 0.85)	There was statistical significant relationship between non-medical use of prescription stimulants and anxiety (*F*(1, 799) = 12.44, *p* < 0.001, η2 = 0.015), stress (*F*(1, 799) = 17.75, *p* < 0.001, η2 = 0.022), but not for depressive symptomatology (*F*(1, 799) = 3.221, *p* = 0.073, η2 = 0.004).
Betancourt et al. (2013) Puerto Rico	Quantitative non-randomized	Age: 21–53 Females: 67.6%	*N* = 252 N_NMPDU_ = 76 (27.6%)	Determine the associations between self-perceived academic load and stress, NMUPD (stimulants, depressants, and sleeping medication), and dietary pattern in college students in Puerto Rico.	Perception of academic load, Perception of stress.	Perception-of-stress scale adapted from the Systemic Cognitive Model of Academic Stress	Prescription medication as a coping strategy: NMUPD (stimulants, depressants, and sleeping medications)	Questions about the use of prescription drugs	Those with higher levels of stress had higher NMUPD (42.1%) than did those with low (26.3%) or moderate (31.6%) stress levels, after controlling for age and sex (*p* = 0.069). NMUPD was significantly associated with stress, after controlling for age and sex (OR = 1.482; 95% C.I. = 1.036, 2.120; *p* = 0.03). No significant association was found between NMUPD and academic load, even after controlling for age and sex (OR = 1.354; 95% C.I. = 0.774, 2.369; *p* = 0.29).
Cabriales et al. (2013) USA	Quantitative non-randomized	*M_age_* = 20.08 (*SD* = 3.96) Females: 59%	*N* = 435 N_NMPDU_ = 29.4% lifetime	Assess lifetime prescription drug misuse rates as well as potential protective and risk factors for misuse in a Hispanic college sample	Depression, Anxiety, Stress.	DASS: depression, anxiety and stress (depression α = 0.95, anxiety α = 0.88, and stress α = 0.92).	Prescription drug use: opioid analgesics, sedatives/tranquilizers, and stimulants, lifetime.	*Ad hoc* questionnaire	Higher anxiety level significantly increased the odds of having ever misused prescription drugs, B = 0.08, OR = 1.08, 95% confidence interval (CI) [1.01, 1.16], *p* < 0.05. Higher depressive symptomatology was significantly associated with lower odds of ever misusing prescription drugs, B = − 0.07, OR = 0.93, 95%, CI [0.88, 0.99], *p* < 0.05
Dussault and Weyandt (2013) USA	Quantitative non-randomized	Age: > 18 Female = 723	*N* = 1,033 *n_NMPSU_* = 204 (19.8%)	Examine whether psychological variables were related to self-reported nonmedical stimulant use.	Depression, Anxiety and stress	Dass-21:depression, anxiety and stress	Misuse of prescription stimulants	SSQ (α = 0.85)	Only anxiety scale was a significant predictor of Non-medical stimulant use, *t*(1022) = 2.472, *p* = 0.014; with those reporting higher rates of anxiety also reporting higher rates of nonmedical stimulant use.
Zullig and Divin (2012) USA	Quantitative non-randomized	Age: 18–25 Females: 69.26%	*N* = 22,783 *n_NMPU_ =* 2,962 (13%) any drug. *n_NMPD_opioid* = 1,913 (8.4%) *n_NMPD_stimulant =* 1,349 (5.9%) *n_NMPD_sedatives =* 944 (4.1%) *n_NMPD_antidepressants =* 685 (3.0%)	Explored the association between general and specific NMPDU, depressive symptoms, and suicidality.	Depressive symptoms and suicidality	Six *ad hoc* mental health questions	Non-medical Prescription Drug Use: antidepressants, painkillers, sedatives, and stimulants, past 12 months.	Ad hoc question (“Within the last 12 months, have you taken any of the following prescription drugs that were not prescribed to you?”)	Those who reported feeling hopeless, sad, depressed, or considered suicide were still between 1.22 and 1.31 more likely to report any NMPDU. Both unadjusted and adjusted comparisons for each of the NMPDU variables suggest that college students who reported NMPDU have significantly greater odds of reporting depressive symptoms and/or suicidality. When the adjusted models were repeated separately by gender, results were more pronounced for females, especially for females who reported painkiller use.
McCauley et al. (2011) USA	Quantitative non-randomized	Age: ≥18 Females: 100%	*N* = 2,000 *N_NMUPD_* = 155 (7.8%)	Examine mental health and other demographic characteristics as potential risk correlates of NMUPD in a national sample of college women	Health and mental health: Lifetime PTSD and MDE, Rape experiences	National Women’s Study (NWS) PTSD and Major Depressive Episode modules, structured interviews	Non-medical use of prescription drugs: tranquilizers, sedatives, stimulants, steroids, and pain medicines.	*Ad hoc* questionnaire	Lifetime MDE remained a significant predictor (OR = 2.14 vs. no MDE), while lifetime PTSD only maintained a trend toward significance (OR = 1.59; *p* = 0.06 vs. no PTSD). Lifetime MDE (OR = 2.67 vs. no MDE; 95% CI [1.74–4.11]) and PTSD (OR = 1.68 vs. no PTSD; 95% CI [1.09–2.58]) were associated with prescription drug misuse.
Teter et al. (2010) USA	Quantitative non-randomized	*M_age_* = 19.9 (*SD* = 2.0) Females: 53.6%	*N* = 3,639 *n_NMUPS_* = 212 (6%)	H1: NMUPS and nonoral routes of NMUPS administration would each be associated with higher rates of depressed mood. Analyze the relationships between other student variables (e.g., gender, race) and depressed mood	Depressed mood	Two-item PHQ-2: depressed mood	NMUPS: Stimulant medication (e.g., Ritalin, Dexedrine, Adderall, Concerta, methylphenidate).	Ad hoc question: “On how many occasions in (a) your lifetime or (b) the past 12 months or (c) the past 30 days have you used the following types of drugs, not prescribed to you? Stimulant medication”	Adjusted odds of depressed mood were over two times greater among frequent monthly NMUPS (adjusted odds ratio [AOR] = 2.3, 95% confidence interval [CI] = 1.01–5.15) and non-oral routes of administration (AOR = 2.2, 95% CI = 1.36–3.70), after controlling for other variables.
Vidourek et al. (2010) USA	Quantitative non-randomized	Females: 58%	*N =* 363 *N_NMPDU_* = 112 (32%)	Does involvement in risky behaviors including sexual behaviors, substance abuse and suicidal ideation differ based on use of NMPDs?	Suicidal ideation	*Ad hoc* questionnaire	Non-Medical Prescription Drug Use: sleeping medication, sedative or anxiety medication, stimulant and pain medication	*Ad hoc* questionnaire	Students who had ever engaged in NMPD use had greater odds of lifetime suicidal ideation (OR = 2.459, CI 95% = 1.168, 5.177) and in considering whether to attempt suicide in the past 12 months (Or = 3.870, CI 95% = 1.109, 13.510), compared to students who had never used NMPD.
Weyandt et al. (2009) USA	Quantitative non-randomized	Females: 255 (71,6%)	*N* = 363 n_NMPS_ = 27 (7,5%)	Explore whether psychological variables and demographic variables were related to nonmedical use of prescription stimulants among college students	Depression and anxiety.	BSI	Misuse of stimulants past 30 days, past 12 months	SSQ: use and misuse of prescription stimulant medications	There is a relationship between stimulant use and degree of psychological distress and internal restlessness. Students who reported higher ratings on the stimulant survey also reported higher ratings of psychological distress There is a statistically significant relationship between self-reported prescription stimulant use and stress (*r* = 0.356, *p* < 0.01)
Ford and Schroeder (2008) USA	Quantitative non-randomized	Age: 15–25 *M_age_* = 21 Female = 61%	*N =* 11,215 *n*_NMPSU_ = 224 (2%) past 30 days. *n*_NMPSU_ = 448 (4%) past year.	Analyze the relationship between academic strain and non-medical use of prescription stimulants	Academic strain, depression	*Ad hoc* questionnaire	Non-medical use of prescription stimulants: in the past year and the past 30 days	*Ad hoc* question	There is no direct connection between academic strain and stimulant use in the past year, the connections is indirect via negative affect (depression). Depression is significantly associated with non-medical prescription stimulant use (Beta = 0.045, *p* < 0.001), in the past year and in the past 30 days (Beta = 0.032, *p* < 0.001). Students who report higher levels of depression are more likely to report the non-medical use of prescription stimulants

*NMPO (Non-medical prescription opioid); NMPM (Non-medical prescription misuse); PDM (prescription drug misuse); POM (Prescription opioid misuse); STB (Suicidal Thoughts and/or Behavior); NMUPD (Non-medical Use of Prescription Drug); PSM (Prescription Stimulant Misuse); NMPS (Nonmedical Use of Prescription Stimulants); OTC (Over the Counter); IUPS (illicit use of prescription stimulants); PHQ-9 (Patient Health Questionnaire-9); GAD-7 (Generalized Anxiety Disorder-7); CES-D (Center for Epidemiological Studies-Depression); PTSD (posttraumatic stress disorder); MDE (major depressive episode); DAST-10 (10-item Drug Abuse Screening Test); PSS (Perceived Stress Scale. 10-item self-report questionnaire); PSS-4 (4-item Perceived Stress Scale); NMPOQ (Non-medical use of prescription opioid questionnaire); NMBM (Lifetime nonmedical use of Benzodiazepine); HADS (Hospital Anxiety and Depression Scale); SF-CESD (Center for Epidemiological Studies Depression Short Form); CESD-R (Center for Epidemiological Studies Depression Scale Revised); SSA (State Social Anxiety Scale); DASS-21 (Depression, Anxiety and Stress Scale-21); BSI-18 (Brief Symptom Inventory – 18); BSI (Brief Symptom Inventory); SSQ (medical and non-medical prescription stimulant medications among college students).

### Depression and psychotropic drug consumption

Several studies have confirmed that the presence of depressive symptoms in university students is related to the consumption of psychotropic drugs, both with medical prescription (Balayssac et al., 2018) and without prescription or with misuse (Balayssac et al., 2018; Benson and Flory, 2017; Ford and Schroeder, 2008; Freibott et al., 2024; Gaume et al., 2024; Hua et al., 2023; Walters et al., 2018; King et al., 2020; Kouros and Papp, 2024; McCauley et al., 2011; Meshesha et al., 2017; Peralta et al., 2016; Tam et al., 2020; Teter et al., 2010; Čanković et al., 2023; Weyandt et al., 2009). Some of these results suggest a significant and direct relationship (Ford and Schroeder, 2008; Freibott et al., 2024; Gaume et al., 2024; Hua et al., 2023; Walters et al., 2018; Kouros and Papp, 2024; Meshesha et al., 2017; McCauley et al., 2011; Tam et al., 2020; Čanković et al., 2023; Weyandt et al., 2009). On the other hand, some studies report no association between depressive symptoms and psychotropic drug consumption (Sousa et al., 2020), or an inverse relationship in the case of misuse of psychotropic drugs, finding that higher depressive symptoms are associated with a lower likelihood of misusing prescribed medications (Cabriales et al., 2013; Verdi et al., 2014).

In terms of the type of psychotropic drug, the relationship between depressive symptoms and consumption is confirmed for opioids (Freibott et al., 2024; Hua et al., 2023; Meshesha et al., 2017; Weyandt et al., 2021; Zullig and Divin, 2012), stimulants (Benson and Flory, 2017; Ford and Schroeder, 2008; King et al., 2020; Teter et al., 2010; Weyandt et al., 2009; Zullig and Divin, 2012), sedatives (Gaume et al., 2024; Hua et al., 2023; Walters et al., 2018; Tam et al., 2020; Čanković et al., 2023; Zullig and Divin, 2012), and antidepressants (Zullig and Divin, 2012).

### Anxiety and psychotropic drug consumption

There is evidence of a relationship between anxiety symptoms and the consumption of psychotropic drugs without medical prescription or with misuse (Balayssac et al., 2018; Cabriales et al., 2013; Freibott et al. (2024); Gaume et al., 2024; Hua et al., 2023; Jeffers et al., 2015; Tam et al., 2020; Verdi et al., 2014; Weyandt et al., 2021), particularly with stimulants (Dussault and Weyandt, 2013; King et al., 2020; Verdi et al., 2014; Weyandt et al., 2009), opioids (Freibott et al., 2024; Hua et al., 2023) ad sedatives (Gaume et al., 2024; Hua et al., 2023). Regarding the relationship with anxiety, a significant and direct association has also been identified (Balayssac et al., 2018; Tam et al., 2020). On the other hand, Walters et al. (2018) report no association between anxiety symptoms and psychotropic drug consumption.

### Stress and psychotropic drug consumption

There is evidence to suggest that stress is associated with the consumption of psychotropic drugs in general (Balayssac et al., 2018; Boulton and O'Connell, 2017), as well as with non-prescription stimulants (Antshel et al., 2021; Schepis et al., 2021). Several studies have indicated a correlation between stress and the use of psychotropic drugs (Bahlaq et al., 2023; Betancourt et al., 2013; Schepis et al., 2021; Verdi et al., 2014). On the other hand, the study by Grant et al. (2018) directly associated non-prescription stimulant use with post-traumatic stress. Also, the study of Sattler (2019), reported that higher levels of stress were associated with a likely use of stimulants (named like Cognitive Enhancement drug use). Finally, the study of Bahlaq et al. (2023) reports a relationship between stimulants misuse and stress symptoms.

### Suicidal behavior and use of psychotropic drugs

In general, studies find a relationship between the use of prescribed anxiolytics and opioids (Davis et al., 2020; Zullig and Divin, 2012), sedatives (Zullig and Divin, 2012), and antidepressants (Zullig and Divin, 2012) with suicidal ideation, attempts, and behavior.

On the other hand, in the study of Vidourek et al. (2010) concludes that students who had never used psychopharmaceuticals without a prescription were more likely to have lifetime suicidal ideation and to contemplate attempting suicide in the past 12 months.

## Discussion

The findings confirm consistent associations between psychotropic drug use and depressive, anxiety, and stress symptoms in university populations. Crucially, non-prescribed use is more strongly linked to anxiety, stress, and depression, whereas prescribed use is more closely associated with suicidal ideation and behavior (particularly involving antidepressants, anxiolytics, and opioids). This dual pattern, evident across the included studies, should frame risk interpretation and guide differential prevention strategies by type of use.

Most of the identified evidence is consistent regarding the relationship between psychotropic medication and depression (Balayssac et al., 2018; Benson and Flory, 2017), anxiety (Balayssac et al., 2018; Tam et al., 2020), and stress (Balayssac et al., 2018; Betancourt et al., 2013; Boulton and O'Connell, 2017) within the university context. However, the primary finding of this systematic review indicates that symptoms related to depression, anxiety, and stress are more strongly associated with non-prescription psychotropic medication use (Antshel et al., 2021; Balayssac et al., 2018; Bahlaq et al., 2023; Cabriales et al., 2013; Dussault and Weyandt, 2013; Freibott et al., 2024; Ford and Schroeder, 2008; Gaume et al., 2024; Grant et al., 2018; Hua et al., 2023; Jeffers et al., 2015; King et al., 2020; McCauley et al., 2011; Meshesha et al., 2017; Peralta et al., 2016; Sattler, 2019; Schepis et al., 2021; Tam et al., 2020; Teter et al., 2010; Verdi et al., 2014; Weyandt et al., 2009, 2021). In this regard, Balayssac et al. (2018) report that students who self-medicate exhibit higher rates of anxiety and depression than their counterparts who consume some form of psychotropic medication, as directed by a medical professional.

In contrast, suicidal ideation and completed suicide were more strongly associated with prescribed psychotropic medication use, particularly antidepressants, anxiolytics, and opioids (Davis et al., 2020; Zullig and Divin, 2012). However, the study of Vidourek et al. (2010) proposed that there was a major odd of suicidal ideation in students that have ever consumed NMPD and that in a prevalence of last year, the same students were more likely to contemplated the idea of suicide.

In relation to the consumption of non-prescription psychotropic medications, the most compelling evidence suggests that these drugs are readily accessible among university students (Hulme et al., 2018; Verdi et al., 2014). In this context, it is evident that measures to enhance awareness among family and friends are essential, as they represent the primary facilitators of non-prescription psychotropic medications (Ford et al., 2020; Hulme et al., 2018; Schepis and Krishnan-Sarin, 2009; Schepis et al., 2019). Furthermore, the consumption of non-prescription psychotropic medications is associated with a number of adverse health outcomes, including overdoses, addiction, and increased demand for addiction treatment. Additionally, there is a correlation between the use of these medications and polydrug use, as well as fatalities [Center for Disease Control and Prevention (CDCP) and National Center for Health Statistics (NCHS), 2017].

In relation to suicidal behavior, multiple studies (Andersen et al., 2023; Choi et al., 2020; Dogan et al., 2016) clearly highlight the role of psychotropic medications. Symptoms of depression have an indirect effect through stress on suicidal ideation (Restrepo et al., 2018), suggesting that academic stressors in students with depression may increase the risk of suicidal behavior. Additionally, several studies have examined the potential adverse effect of psychotropic medication use on suicidal behavior. Khan et al. (2022) emphasized the relationship between the use of Zolpidem and an increased risk of suicide, finding a dose-dependent association.

As previously mentioned, the role of stress in relation to psychotropic drug use among university students may extend beyond a mere association between the two. Various studies have linked stress and high academic demands with psychotropic drug use (Betancourt et al., 2013; Schepis et al., 2021), motivated by the desire to enhance academic performance, increase concentration and alertness, and facilitate studying (Betancourt et al., 2013; Cook et al., 2021; Gallucci et al., 2014, Gallucci and Martin, 2015; Ghandour et al., 2012; Molloy et al., 2019; Ponnet et al., 2015; Schepis et al., 2021; Yomogida et al., 2018; Zahavi et al., 2023). In fact, the primary motivation for the use of psychotropic drugs without a prescription was academic (Schepis et al., 2021). This means that the structure of the university academic system, the demands placed on students, and their lack of active coping resources place them at greater risk of using psychotropic drugs without a prescription. As Schepis et al. (2021) argue, education extends beyond academic knowledge, with universities serving as a socialization context for promoting holistic and healthy education.

### Sex and LGBTQ+ disparities

Several studies show a higher likelihood of psychotropic drug use associated with psychological distress symptoms among women (e.g., higher odds of use linked to depression and/or suicidality; effects particularly pronounced for analgesics) (Zullig and Divin, 2012).

In national female samples, major depressive episodes and posttraumatic stress disorder were associated with nonmedical use of psychotropic drugs (McCauley et al., 2011). Among LGBTQ+ students, there were indications of a higher prevalence of non-prescribed use to relieve anxiety compared with the heterosexual population (Tam et al., 2020). Conversely, some studies suggest that men with anxiety symptoms may exhibit higher consumption, although without statistically significant differences compared with women in certain samples (Benotsch et al., 2014).

Taken together, the evidence points to meaningful disparities by sex and sexual orientation that should be considered when designing preventive and institutional interventions.

### Proposals from healthy university campuses

Based on the findings of the present systematic review, Healthy University Campuses provide an ideal framework for implementing measures from an integral, preventive, and structural perspective to address the use of psychotropic drugs, both prescribed and non-prescribed, linked to psychological distress among university students. It is proposed that Healthy Campus initiatives strengthen actions aimed at informing and raising awareness about the risks associated with the misuse and non-medical use of psychotropic drugs, as well as promoting a more comprehensive institutional response to student psychological distress. In this regard, it is essential to implement ongoing informational campaigns that highlight the risks of non-prescribed psychotropic drug use and its association with symptoms of anxiety, depression, stress, and suicidal ideation. These campaigns should be complemented by the inclusion of educational content on safe medication use and healthy coping strategies in the students’ cross-curricular training programs.

Additionally, it is necessary to offer early detection services, brief interventions, and referral pathways within the university setting. To achieve this, it is recommended to establish accessible psychological support services on campus that enable early identification of emotional distress, problematic drug use, or suicidal ideation. These services should include brief, non-stigmatizing interventions and ensure timely and effective referral to primary care or external mental health services when appropriate.

Moreover, fostering students’ psychosocial well-being through the active promotion of healthy lifestyles is essential. This involves creating university environments that prioritize a balance between academic performance and key aspects such as rest, physical activity, healthy eating, and the development of meaningful social relationships. These actions should be embedded within an institutional policy that promotes well-being in a transversal and sustained manner.

Finally, there is a need to critically reflect on the current academic model. Universities must examine the structural and cultural factors that act as chronic stressors, such as competitiveness, performance pressure, and academic overload. Within this context, it is suggested that institutions explore reforms that encourage an academic culture based on meaningful learning and cooperation, as part of a broader strategy to prevent emotional distress among students. In this sense, Tam et al. (2020) emphasize the importance of analyzing the relationship between mental health and psychotropic drug use from a psychosocial and environmental perspective.

### Limitations

One of the main limitations of the present systematic review is that does not consider sex as a mediating variable. Although several studies suggest subgroup differences (e.g., higher likelihood of psychotropic drug use among women (especially analgesics) and a possible higher rate of non-prescribed use to manage anxiety among LGBTQ+ students), the heterogeneity of definitions and measures, sample sizes, and the low power of subgroup analyses, together with findings that are not always consistent (e.g., higher consumption among men with anxiety without clear statistical differences), limit the strength of the inferences; therefore, the results should be interpreted as exploratory. Future studies should delve into the sex as a mediating variable between nonmedical psychotropic consumption and depression, stress, anxiety, suicidal ideation and suicide.

On the other hand, the research conducted is largely focused geographically on the United States, which hinders the generalization of the results to other countries. Similarly, various university models, as well as different economic, social, demographic, and environmental factors in each country, may influence the relationship between mental health and psychotropic drug consumption. Therefore, future studies should delve into these issues further.

In addition, the analysis of the present study has led to the emergence of several questions that future research can address and that Healthy Campuses should reflect upon. What is the role of coping styles in the use of non-prescribed psychotropic drugs in the context of psychological distress among university students? Should university students be provided with training in adaptive coping skills and strategies during their early years? It is also necessary to consider whether Healthy Campuses provide sufficient guidance and mental health support resources for the university community. What measures should be implemented to address contextual and academic determinants from an environmental perspective?

## Conclusion

The results of this systematic review demonstrate a significant relationship between the use of psychotropic drugs, both prescribed and non-prescribed, and the presence of symptoms of depression, anxiety, stress, suicidal ideation, and suicidal behavior among university students. Specifically, non-medical use of psychotropic drugs is primarily associated with symptoms of psychological distress such as anxiety, stress, and depression, while prescribed use is more closely linked to suicidal ideation and completed suicide.

Moreover, disparities are observed by sex and within the LGBTQ+ population. Women show a higher likelihood of use linked to depression and suicidal behavior, and there are signs of greater non-prescribed use among LGBTQ+ students for coping with anxiety. These findings reinforce the need for intersectional approaches in both research and practice.

These findings underscore the need to adopt an integral, contextual, and preventive approach to address psychotropic drug use within the university setting. The Healthy University Campus framework offers a strategic opportunity to develop policies and programs that not only inform about the risks of non-medical use, but also promote psychosocial well-being, early detection of emotional distress, and the transformation of structural factors that contribute to such issues.

Finally, the importance of continuing to investigate this phenomenon from an intersectional and multilevel perspective is highlighted, taking into account individual, social, academic, and cultural variables. Only through a coordinated, evidence-based institutional response will it be possible to reduce problematic psychotropic drug use in universities and ensure an environment that fosters students’ mental health.

## Data Availability

The raw data supporting the conclusions of this article will be made available by the authors, without undue reservation.
